# A Tutorial on Cognitive Diagnosis Modeling for Characterizing Mental Health Symptom Profiles Using Existing Item Responses

**DOI:** 10.1007/s11121-022-01346-8

**Published:** 2022-02-03

**Authors:** Zhengqi Tan, Jimmy de la Torre, Wenchao Ma, David Huh, Mary E. Larimer, Eun-Young Mun

**Affiliations:** 1 Department of Biostatistics and Epidemiology, University of North Texas Health Science Center, 3500 Camp Bowie Blvd, Fort Worth, TX 76107 USA; 2 Faculty of Education, The University of Hong Kong, Hong Kong, China; 3 College of Education, The University of Alabama, Tuscaloosa, AL USA; 4 School of Social Work, University of Washington, Seattle, WA USA; 5 Department of Psychiatry and Behavioral Sciences, University of Washington, Seattle, WA USA; 6 Department of Health Behavior and Health Systems, University of North Texas Health Science Center, Fort Worth, TX USA

**Keywords:** CDM, Diagnostic classification model, DCM, IRT, Assessment, Classification, Co-occurring symptom profiles, Research domain criteria

## Abstract

In research applications, mental health problems such as alcohol-related problems and depression are commonly assessed and evaluated using scale scores or latent trait scores derived from factor analysis or item response theory models. This tutorial paper demonstrates the use of cognitive diagnosis models (CDMs) as an alternative approach to characterizing mental health problems of young adults when item-level data are available. Existing measurement approaches focus on estimating the general severity of a given mental health problem at the scale level as a unidimensional construct without accounting for other symptoms of related mental health problems. The prevailing approaches may ignore clinically meaningful presentations of related symptoms at the item level. The current study illustrates CDMs using item-level data from college students (40 items from 719 respondents; 34.6% men, 83.9% White, and 16.3% first-year students). Specifically, we evaluated the constellation of four postulated domains (i.e., alcohol-related problems, anxiety, hostility, and depression) as a set of attribute profiles using CDMs. After accounting for the impact of each attribute (i.e., postulated domain) on the estimates of attribute profiles, the results demonstrated that when items or attributes have limited information, CDMs can utilize item-level information in the associated attributes to generate potentially meaningful estimates and profiles, compared to analyzing each attribute independently. We introduce a novel visual inspection aid, the lens plot, for quantifying this gain. CDMs may be a useful analytical tool to capture respondents’ risk and resilience for prevention research.

**Supplementary Information:**

The online version contains supplementary material available at 10.1007/s11121-022-01346-8.

## Introduction

In prevention research, a prevailing approach to assessing mental health problems has been to estimate their symptom severity based on their clinical taxonomy and separately determine whether that severity exceeds a pre-defined threshold for screening and intervention purposes, without accounting for potentially relevant information of how symptoms covary or interact. In other words, the existing measurement approach focuses on quantifying general trait levels (e.g., severity of depression or reading ability) via factor analysis or item response theory (IRT) analysis. However, this standard measurement practice may overlook the presence of potentially significant covariations or clusters of variations among related items in terms of symptom presentations. This tutorial article presents cognitive diagnosis models (CDMs) as an alternative approach when item-level measurement data is available and demonstrates its application to the assessment of mental health problems among college students.

CDMs were developed to identify the presence or absence of *symptom clusters* defined by a set of items (Haertel, 1989) and to better distinguish individuals with similar scores by differentiating one *symptom profile* (i.e., a set of symptom clusters) from another based on an item-by-item analysis. CDMs probe across items to classify respondents based on their symptom profiles. In the current paper, we refer to the individual symptom clusters as *attributes.* Attributes are typically assumed to be binary with two statuses—presence or absence. Further, *attribute profiles* reveal which symptom profiles the respondents concurrently have within the domain of interest being considered. In the following sections, we first provide the general description of CDMs along with their features, followed by an application of CDMs to a real data example. We then discuss the advantages and implications of utilizing CDMs as an assessment tool for prevention research.

### Model Formation in CDMs

CDMs are latent variable models that can be used to characterize attribute profiles based on the presence or absence of multiple postulated attributes. In the motivating data example, those attributes correspond to latent mental health problems (e.g., anxiety, depression, hostility, alcohol-related problems), but in other applications, they could represent skills, cognitive processes, or solution strategies. There are different formulations of CDMs (de la Torre et al., 2018; von Davier & Lee, 2019), and in this tutorial, we focus on the generalized deterministic, input, noisy, “and” gate model (G-DINA model; de la Torre, 2011). The G-DINA model provides a general framework for formulating saturated and reduced CDMs, comparing models and evaluating model fit at the item and scale (or test) levels, and empirically validating the postulated associations between items and attributes.

The implementation of the G-DINA model, like many CDMs, requires constructing a Q-matrix (Tatsuoka, 1983), which specifies whether each assessed item is associated with one or more pre-defined attributes (e.g., anxiety and depression). Thus, a Q-matrix requires the explicit use of substantive domain knowledge to specify which items are related to which attributes (Leighton et al., 2004). A Q-matrix is typically constructed a priori based on expert judgment, clinical theory, or empirical research findings (de la Torre, 2008). A Q-matrix is a matrix of ones and zeros, where each row corresponds to a specific item, and each column corresponds to a specific attribute. Thus, an element within the Q-matrix indicates whether a specific item measures a given attribute (1 = yes; 0 = no). For example, a 40-item inventory across four attributes requires a 40-by-4 matrix of 1 s and 0 s, defining how the 40 items are associated with each of the four attributes. In addition, the individual respondent’s attribute profile is estimated by fitting CDMs to the data. Each respondent has an associated attribute profile indicating whether each attribute is present (= 1) or absent (= 0). In a scenario with four different attributes, a respondent can belong to one of 2^4^ (= 16) possible attribute profiles. For example, if a respondent’s profile is “0 0 0 0,” the respondent does not have any of the four attributes, whereas the pattern “0 1 0 1” indicates the respondent has the second and the fourth attributes.

The item response function of the saturated G-DINA model describes the probability that an item is endorsed when modeling the main effects of the attributes in combination with all possible interaction effects among all the attributes. However, reduced CDMs can often be derived based on specific assumptions about how the underlying attributes affect item endorsement. For example, all interaction effects can be constrained to zero, leaving only additive main effects (i.e., referred to as an additive CDM; de la Torre, 2011), which assumes that each attribute contributes independently and additively to the probability of endorsing items. Using a logit and a log link function, with the same additive assumption, we can obtain the linear logistic model (LLM; Maris, 1999) and the reduced reparameterized unified model (RRUM; Hartz, 2002), respectively (for more detailed information regarding reduced CDMs, see de la Torre, 2011). These reduced CDMs are compared against the saturated model to determine whether a more parsimonious model can be selected and interpreted (Ma et al., 2016). Hence, evaluating the absolute and relative model fit statistics from competing CDMs is critical when selecting a final set of models. The absolute model fit of CDMs can be evaluated using the limited information root mean square error approximation (RMSEA_2_; Maydeu-Olivares & Joe, 2014), with RMSEA_2_ < .05 indicating adequate fit. With respect to relative fit, the Wald test can be used for each item to determine whether a reduced model can be used instead of the saturated (or full) G-DINA model without a significant drop in fit between the model and the data. The relative model fit can also be evaluated using Akaike Information Criterion (AIC) and Bayesian Information Criterion (BIC), with a smaller absolute value indicating a better fit to the data. We note that the evaluation of model fit statistics for CDMs is an active area of research (Han & Johnson, 2019).

Thus far, we have discussed the measurement component of CDMs at the item level (e.g., G-DINA, LLM, RRUM). The relationships among attributes (i.e., the joint distributions) also need to be examined to determine how the attributes relate to one another, which is often referred to as the *structural component of the model*. The current paper uses the log-linear model as the structural component, as in Xu and von Davier (2008), since the log-linear model can better elucidate the relationships among attributes (Henson et al., 2009). Once the structural model is selected, the parameters of the selected model can be estimated using the expectation–maximization algorithm (de la Torre, 2011; Xu & von Davier, 2008). The reliability of classification results can be evaluated using the test-level classification accuracy index and attribute-level classification accuracy index (Wang et al., 2015).

### Model Validation and Tools

**Fig. 1:**
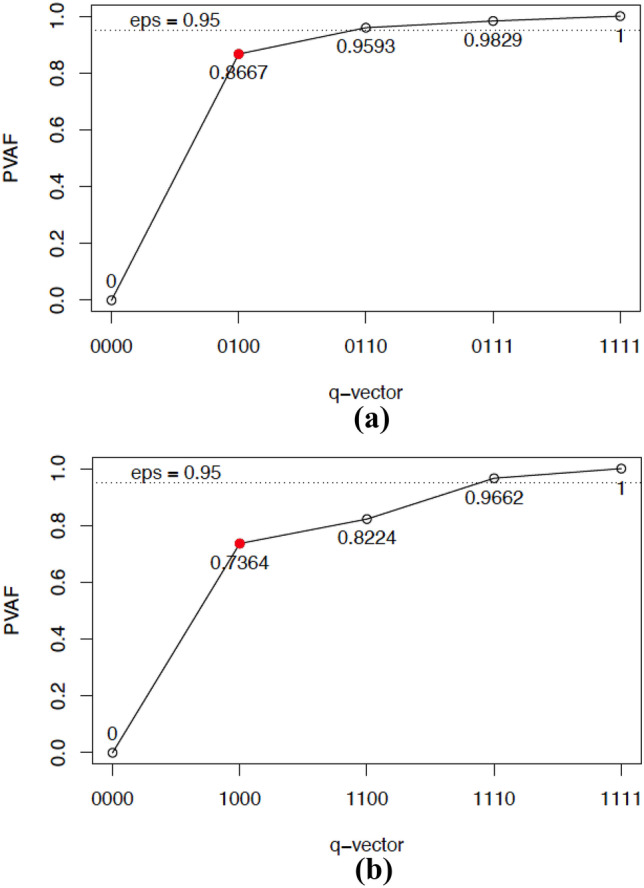
The mesa plots of item 29 “Feeling so restless you couldn’t sit still” (**a**; top) and item 10 “Had withdrawal symptoms, that is, felt sick because you stopped or cut down on drinking?” (**b**; bottom). The q-vector represents the best 1-, 2-, 3-, and 4-attribute specification for the item. PVAF, proportion of variance accounted for. The filled circle indicates the original q-vector. eps, epsilon, a default cutoff value for PVAF

Having detailed all the major steps of CDMs, we return to the step of formulating an initial Q-matrix, which can be empirically validated using the data. During the validation phase of the Q-matrix, expert judgment may be reconciled with empirical evidence (i.e., the observed data) in determining the final attribute specification for each item. This validation procedure involves searching for the simplest attribute specification of an item, which is evaluated using the G-DINA discrimination index (GDI; de la Torre & Chiu, 2016). An acceptable proportion of variance accounted for (PVAF) relative to the maximum GDI of the item (de la Torre & Chiu, 2016) can be visualized in a *mesa plot* (de la Torre & Ma, 2016). The mesa plot is a line graph, where the *x*-axis shows the possible item-attribute specifications (known as the q-vector), and the *y*-axis gives the corresponding PVAFs. It is conceptually similar to a scree plot used in factor analysis, and the location where the edge of a mesa can be seen is considered the correct attribute specification for the item. Figure 1 shows a mesa plot for two items from the motivating data example described in the next section.

**Fig. 2:**
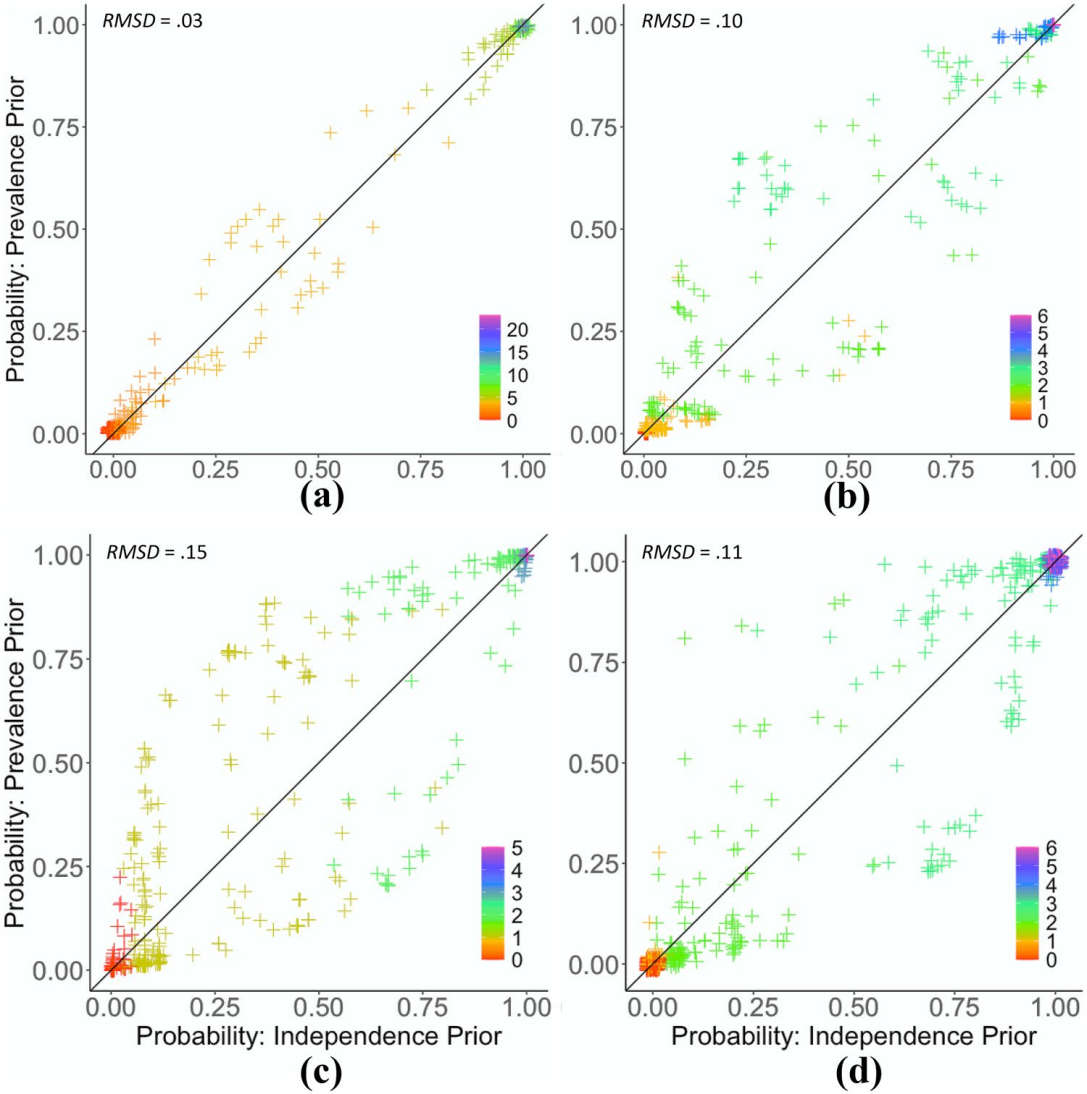
Lens plots of the estimated attribute probabilities estimated using the independence prior (horizontal axes) and the prevalence prior (vertical axes) for alcohol-related problems (**a**; top left), anxiety (**b**; top right), hostility (**c**; bottom left), and depression (**d**; bottom right). Diagonal lines are where *y* = *x*. All *Y*- and *X*-axes share the same unit and scale. The legend indicates the number of items endorsed within each attribute. Small random noise was added to help distinguish overlapping data points. RMSD, root mean squared differences

In this tutorial, we introduce an additional new visual aid, the lens plot, to graphically check the extent to which the presence of other attributes helps refine the estimation of the probability of a particular attribute (see Fig. 2). The lens plot visually shows the difference in posterior probabilities of an attribute profile for all individuals under two different prior assumptions. The two different assumptions are that the attributes are (1) associated with other attributes (i.e., joint prevalence prior) and (2) independent of one another (i.e., independence prior). The *joint prevalence prior* is based on the CDM analysis, and accounts for attribute co-occurrences (prevalence of the attribute profiles). The *independence prior* maintains the *individual attribute* prevalences (i.e., *marginal probabilities*) of the joint prevalence prior; but assumes that the attributes are independent. The posterior probabilities based on the joint prevalence prior (henceforth, prevalence prior) can be interpreted as the estimated probabilities of having an attribute while accounting for the impact of other attributes. Points falling on the diagonal line of the lens plot indicate that a joint analysis taking into account attribute co-occurrences provides no additional gain. Points above and below the diagonal line suggest increased and decreased estimated probabilities, respectively. Hence, a lens with a wider optical center indicates that taking into account co-occurring attributes incorporates more information than examining them as independent attributes. The overall information input from other attributes on the probability estimation of a given attribute *k* can be quantified by the root of the mean squared differences (RMSD) between the two posterior probabilities estimated based on the independence and prevalence priors. For any given point $\left({P}_{ik}^{(in)},{P}_{ik}^{(pr)}\right)$, RMSD can be defined as follows:

$${{RMSD}}_{k}=\sqrt{\frac{\sum_{i}{\left({P}_{ik}^{(in)}-{P}_{ik}^{(pr)}\right)}^{2}}{N}},$$

where ${P}_{ik}^{(in)}$ and ${P}_{ik}^{(pr)}$ denote the probabilities that person *i* has attribute *k* based on the independence and prevalence priors, respectively. Hence, RMSD provides a quantitative summary of the differences between all estimated posterior probabilities based on two different priors.

In the following section, we describe the motivating data example and demonstrate the use and interpretation of CDMs for generating attribute profiles based on existing measures of mental health symptoms for college students using the R package *GDINA* (Ma & de la Torre, 2020). Annotated computer code in R for demonstrating the use of CDMs, along with example data, can be accessed in the online repository (https://doi.org/10.17632/97bzg6z28h.1; Tan et al., 2022).

## Data Application

### Respondents

Data come from 835 respondents (35.3% men, 81.6% White, and 17.0% first-year students) who originally participated in the Motivating Campus Change Multisite Study (MC^2^; Larimer et al., 2007; see Mun et al., 2015). The MC^2^ study was a large, multisite, and multicohort longitudinal prevention study that evaluated three stepped-care interventions across three university campuses located in the northwestern USA. In the present study, we utilize baseline item-level data from 719 individuals (34.6% men, 83.9% White, 16.3% first-year students) without missing item responses, drawn from one of the campus sites.

### Measures

#### Alcohol-Related Problems

Alcohol-related problems were assessed by the Rutgers Alcohol Problem Index (RAPI; White & Labouvie, 1989), a widely used measure assessing alcohol-related problems among adolescents and young adults. The RAPI is a 23-item, self-report measure that assesses the frequency of alcohol-related problems using an ordinal scale ranging from 0 (never) to 4 (more than 10 times). In the current study, higher scores indicate more negative consequences in the past 3 months. The psychometric properties of the RAPI have been extensively studied (e.g., Neal et al., 2006). Previous studies indicate that a unidimensional, two-parameter logistic item response theory model generally fits data on alcohol-related problems well (Huo et al., 2015; Neal et al., 2006). Cronbach’s alpha for the RAPI with the current sample was .91 (for item mapping information, see Supplemental Table 1).

#### Psychological Symptoms

Anxiety, hostility, and depression were assessed by the Brief Symptom Inventory (BSI; Derogatis, 1975), a widely used assessment to identify clinically relevant psychological symptoms. The BSI is a 53-item self-report measure designed to reflect the typical symptomatology of people experiencing psychiatric problems (Derogatis, 1975). In the current study, all questions shared the same question stem, “During the past 2 weeks, how much were you distressed by,” and they were followed by specific symptoms, such as “nervousness or shakiness” for the anxiety subscale. Each item was answered on a 5-point Likert-type scale ranging from 0 (not at all) to 4 (extremely).

In the current study, we focused on anxiety, hostility, and depression because these attributes tend to co-occur and represent the two most typical developmental pathways of alcohol problems – internalizing and externalizing (Steele et al., 1995; Zucker, 2006). Therefore, these three subscales of the BSI were selected in the current study. Cronbach’s alphas for the anxiety (6 items), hostility (5 items), and depression (6 items) subscales in the current sample were .86, .77, and .90, respectively (for item mapping information, see Supplemental Table 1).

### Data Preparation

We dichotomized the original responses into non-zero (= 1) and zero (= 0, “none” from the RAPI and “not at all” for the BSI) responses. Measures of alcohol-related problems, including the RAPI items, are commonly dichotomized in substantive research since any occurrence of alcohol-related problems within a time frame is more important than how frequently they occurred (Martens et al., 2007). Furthermore, item response distributions suggested dichotomization was reasonable since there was minimal variation in non-zero responses, with more than 86% of the respondents endorsing the majority of items with zero responses. We conducted an additional sensitivity analysis using the *sequential* G-DINA model (Ma & de la Torre, 2016) on the original polytomous responses, which yielded results highly similar to those from the analysis of dichotomized responses (under different response category sizes, the concordance of the profiles estimated from the two models ranged from .80 to 1.00; see details in Supplemental Table 2). Although the sequential G-DINA model was developed for items that involve sequential steps, Ma and de la Torre (2016) showed that when all response categories are assumed to have the same q-vector, the sequential G-DINA model is equivalent to the nominal response diagnostic model and thus is sufficiently flexible for polytomous response data in the present application.

We analyzed an inventory of 40 items that assessed the following four attributes: alcohol-related problems (attribute 1), anxiety (attribute 2), hostility (attribute 3), and depression (attribute 4). We imposed the monotonicity constraint when fitting the CDMs to ensure that having an additional attribute would not lead to a lower probability of endorsing the item. The following section details the CDM analysis, from the Q-matrix construction and validation to the model estimation and evaluation (see Table 1 for a summary of the essential CDM steps).

**Table 1:** Essential steps of CDMs

Step	Purpose
Q-matrix construction and validation	
1. Define the initial Q-matrix	Domain experts to identify how each item is related to attributes
2. Validate the Q-matrix	To validate whether the Q-matrix fit the data through examining the model fit of the saturated model and the mesa plots and proportions of variance accounted for (PVAF) for all items
Model estimation and evaluation	
3. Model selection	To determine whether reduced models can replace the saturated model (at both item and attribute levels)
4. Item parameter estimation	To estimate the parameters of each item to understand item characteristics
5. Cross-attribute impact evaluation	To evaluate the cross-attribute impact on attribute probability estimation through the lens plot and the corresponding root mean squared differences (RMSD)
6. Classification accuracy evaluation	To evaluate the test-level classification accuracy (i.e., attribute profiles) and attribute-level classification accuracy

## Results

### Q-Matrix Specifications

Two domain experts discussed and examined all items and their associated attributes to construct an initial Q-matrix (see Table 2). The general G-DINA model was fitted to the data to validate the initial Q-matrix empirically. All mesa plots were inspected to examine whether possible modifications to the q-vector are desirable for items. Due to the data-driven nature of this step, domain experts’ further review and arbitration were necessary to arrive at the final Q-matrix.

**Table 2:** Q-matrix: linking items with mental health symptoms

Item	Attribute					Attribute			
	AP	AN	HO	DE	Item	AP	AN	HO	DE
1	1	0	0	0	21	1	0	1	0
2	1	0	0	0	22	1	0	0	0
3	1	0	0	0	23	1	0	0	0
4	1	0	0	0	24	0	1	0	0
5	1	0	0	0	25	0	1	0	0
6	1	0	0	0	26	0	1	0	1
7	1	0	0	0	27	0	1	0	1
8	1	0	0	0	28	0	1	0	1
9	1	0	0	0	29	0	1	1	0
10	1	0	0	0	30	0	0	1	0
11	1	0	0	0	31	0	0	1	0
12	1	0	0	0	32	0	0	1	0
13	1	0	0	0	33	0	0	1	0
14	1	0	0	0	34	0	0	1	0
15	1	0	0	0	35	0	0	0	1
16	1	0	0	0	36	0	0	0	1
17	1	0	0	0	37	0	0	0	1
18	1	0	0	0	38	0	0	0	1
19	1	0	0	0	39	0	0	0	1
20	1	0	0	0	40	0	0	0	1

1 = indicates that an item measures the attribute; 0 = indicates an item does not. Underlined elements indicate that they were changed from zero to one following the Q-matrix validation phase

*AP* alcohol-related problems, *AN* anxiety, *HO* hostility, *DE* depression

For example, item 29 (“Feeling so restless you couldn’t sit still”) was initially associated only with the attribute anxiety. The mesa plot for this item (see Fig. 1a) indicated that modifying the Q-matrix to associate item 29 with the attribute hostility would increase the PVAF by .093. Domain experts agreed with this suggestion, and thus, the initial Q-matrix was modified accordingly. In the case of item 10 (“Had withdrawal symptoms, that is, felt sick because you stopped or cut down on drinking?”), the mesa plot suggested an association with the attributes anxiety and hostility, in addition to its initial attribute, alcohol-related problems. However, after carefully reviewing the attribute-related context for the item, the domain experts rejected the suggestion because associating the additional attributes to the item could not be substantively justified, despite the potential increase of .230 in PVAF. Note that in this application, greater weight was placed on the substantive interpretation of the item-attribute association than the traditional default epsilon (eps) cutoff of .95, which can be suboptimal (Nájera et al., 2020).

### Model Comparison and Model Fit

After determining the final Q-matrix, we fit the saturated G-DINA model to the data and examined whether a reduced model could provide an equally acceptable fit for multi-attribute items. The most appropriate CDM for each multi-attribute item (i.e., A-CDM: item 29; RRUM: items 21 and 28; and LLM: items 26 and 27) was selected based on the Wald test. Specifically, in comparing the saturated and Wald-selected models, both models showed adequate absolute fit with an RMSEA_2_ < .05 (Maydeu-Olivares & Joe, 2014). However, the AIC and the likelihood ratios test (LRT) preferred the reduced models (saturated model vs. selected reduced model: AIC: 20,039.12 vs. 20,032.66, LRT: 3.54, *df* = 5, *p* = .62), which subsequently became the basis for selecting the structural component of the model.

In conjunction with the Wald-test-selected CDMs, the saturated structural component of the model containing all main effects and possible 2-way, 3-way, and 4-way interactions was also estimated. We further examined whether reduced models (e.g., models with main effects and lower-order interactions) could provide an equally acceptable fit. The LRT showed that the homogeneous association model (i.e., a log-linear model with up to two-way interactions) fit the data as well as the saturated model (LRT: 7.22, *df* = 5, *p* = .20) and was subsequently chosen.

### Parameter Estimates of Items

Table 3 summarizes the item-level parameter estimates obtained from the CDM analysis for the 40 items. Thirty-five items measuring a single attribute had two parameters: the item endorsement probability of respondents for whom the attribute was absent (= 0) or present (= 1). For example, 29% of individuals for whom the depression attribute was absent endorsed item 37, whereas 96% of individuals for whom the depression attribute was present endorsed the same item. For the remaining five items that measured two attributes, endorsement rates were estimated for those who had none of the attributes (“0 0”), had one of the two attributes (“0 1” or “1 0”), and had both attributes (“1 1”). The latter two endorsement rates show the differential impact of a specific attribute on a given item. For example, 36% of the individuals with only the anxiety attribute were expected to endorse item 26. However, when both anxiety and depression attributes were present, most individuals (83%) endorsed item 26.

**Table 3:** Parameter estimates of items: endorsement rate conditional on attributes

		Attribute Status					Attribute Status				
Item	Attribute	0	1	Disc	Item	Attribute	0		1		Disc.
1	AP	.06	.50	.44	23	AP	.00		.22		.22
2	AP	.02	.38	.36	24	AN	.11		.75		.64
3	AP	.03	.39	.36	25	AN	.01		.61		.60
4	AP	.07	.30	.23	30	HO	.35		.94		.59
5	AP	.05	.39	.34	31	HO	.02		.44		.42
6	AP	.16	.73	.57	32	HO	.02		.34		.32
7	AP	.01	.08	.07	33	HO	.01		.46		.45
8	AP	.04	.43	.39	34	HO	.03		.42		.39
9	AP	.04	.31	.27	35	DE	.01		.31		.30
10	AP	.00	.09	.09	36	DE	.29		.92		.63
11	AP	.12	.49	.37	37	DE	.29		.96		.67
12	AP	.02	.33	.31	38	DE	.10		.80		.70
13	AP	.03	.38	.35	39	DE	.05		.69		.64
14	AP	.07	.45	.38	40	DE	.03		.64		.61
15	AP	.03	.35	.32			Attribute Combination				
16	AP	.01	.20	.19	Item	Attributes	0 0	0 1	1 0	1 1	Disc.
17	AP	.03	.34	.31	21	AP-HO	.13	.18	.61	.86	.73
18	AP	.00	.16	.16	26	AN-DE	.03	.19	.36	.83	.80
19	AP	.02	.38	.36	27	AN-DE	.22	.55	.67	.90	.68
20	AP	.03	.33	.30	28	AN-DE	.00	.01	.22	.50	.50
22	AP	.01	.28	.27	29	AN-HO	.10	.54	.34	.77	.67

0 = the attribute is absent; 1 = the attribute is present; 0 0 = both attributes are absent; 0 1 = only the second attribute is present; 1 0 = only the first attribute is present; and 1 1 = both attributes are present

*AP* alcohol-related problems, *AN* anxiety, *HO* hostility, *DE* depression, disc. discrimination index

For items associated with a single attribute, the difference in the baseline endorsement probabilities of respondents for whom the attribute was absent vs. present represents the item’s power to distinguish individuals with the attribute from those without. This difference can be viewed as the discrimination index of the item (de la Torre, 2008). For the attributes of alcohol-related problems, anxiety, hostility, and depression, the most discriminating items were items 6, 24, 30, and 38, which had discrimination index scores of .57, .64, .59, and .70, respectively. With a mean discrimination index of .39, the 35 single-attribute items, as a whole, can be considered as adequately discriminating the respondents.

The remaining five two-attribute items at the bottom section of Table 3 represent all pairwise combinations of the two attributes in the inventory. The approximate discrimination index of these items can be calculated as the difference between the baseline endorsement probabilities of respondents with both attributes vs. neither attribute. For example, item 26 with a high discrimination index score of .80 distinguished those with neither attribute from those with both the anxiety and depression attributes.

### Structural Model

Table 4 presents the parameter estimates of the log-linear model with homogeneous associations for the structural component of the CDM. This model suggests that the conditional relationship between any pair of attributes is the same across different combinations of the remaining attributes (i.e., no three-way interactions). Note that the alcohol-related problem attribute was not significantly associated with other attributes, whereas anxiety, hostility, and depression attributes exhibited statistically significant homogeneous associations (see Table 4). For example, the attributes depression and hostility were more likely to be co-present (estimate = 3.03, odds ratio = 20.70).

**Table 4:** Parameter estimates of the selected structural model

Variable	Coefficient	Standard error	*p*-value
Intercept	5.74	0.06	< 0.01
Alcohol-related problems	−1.72	0.20	< 0.01
Anxiety	−2.48	0.37	< 0.01
Hostility	−2.68	0.32	< 0.01
Depression	−2.41	0.43	< 0.01
Alcohol-related problems × anxiety	0.32	0.34	0.349
Alcohol-related problems × hostility	0.41	0.42	0.321
Alcohol-related problems × depression	0.70	0.41	0.086
Anxiety × hostility	1.17	0.55	0.033
Anxiety × depression	2.11	0.51	< 0.01
Hostility × depression	3.03	0.46	< 0.01

All coefficients are unstandardized lambda estimates from a log-linear model

### Attribute Prevalence and Classification Accuracy

Fitting CDMs to the response data also yields an estimated prevalence of each mental health symptom profile. The four mental health symptoms can produce up to 16 unique profiles. As can be seen in Table 5, the four most prevalent profiles (refer to the second to last column) were those with none of the attributes (43.8%), the last three attributes (12.6%), all of the attributes (8.8%), and the first attribute only (7.7%). Overall, 38.4% of the respondents exhibited profiles with two or more mental health problems. The estimated prevalence of each profile produced by the prevalence prior and the independence prior is provided in the last two columns of Table 5, for comparison purposes.

**Table 5:** All attribute profiles and their estimated prevalence based on two different priors: the prevalence prior and the independence prior

Attribute profile	Attribute				Prevalence prior (%)	Independence prior (%)
	AP	Anxiety	Hostility	Depression		
1	0	0	0	0	43.77	36.81
2	1	0	0	0	7.74	7.04
3	0	1	0	0	3.29	5.03
4	0	0	1	0	2.91	5.42
5	0	0	0	1	3.91	7.14
6	1	1	0	0	0.97	1.49
7	1	0	1	0	0.60	1.34
8	1	0	0	1	1.08	1.92
9	0	1	1	0	0.91	1.97
10	0	1	0	1	2.69	4.60
11	0	0	1	1	5.24	5.44
12	1	1	1	0	0.43	0.93
13	1	1	0	1	1.58	2.36
14	1	0	1	1	3.49	3.54
15	0	1	1	1	12.61	8.98
16	1	1	1	1	8.78	5.99
Attribute (%)	24.67	31.26	34.98	39.38	100.00	100.00

1 = attribute present; 0 = attribute absent

*AP* alcohol-related problems

The overall attribute profile classification accuracy was .85, indicating that 85% of the respondents could be correctly classified on all four mental health problems. At the individual attribute level, the classification accuracy indices were .97, .95, .94, and .96 for alcohol-related problems, anxiety, hostility, and depression, respectively. These results indicate high classification accuracy at the profile and attribute levels.

### Lens Plot and RMSD

To graphically check the impact of using the joint prevalence of the attributes (i.e., prevalence prior) in estimating an attribute probability, we examined lens plots. Figure 2a shows a thin optical center among the four panels, indicating that this attribute drew the least information from other attributes. Furthermore, very few points could be seen in the off-diagonal quadrant corners of the lens plot, indicating the two probability estimates disagreed only on a very few cases (using a probability of .5 as an empirical cutoff point for having an attribute). In contrast, Fig. 2c shows a thick optical center, indicating that the choice of prior resulted in different probability estimates, hence a large number of classification disagreements. The color legend in Fig. 2 indicates the number of endorsed items for each respondent (see Fig. 2 legends). The optical center of the lens plot was mainly composed of the respondents who endorsed one or two items of the attribute hostility, for example, suggesting that the use of the prevalence prior helped identify the presence of the attribute hostility for those who endorsed one or two items. The overall impact of considering other attributes on the probability estimation of a given attribute was quantified, resulting in RMSDs = .03, .10, .15, and .11, respectively, for alcohol-related problems, anxiety, hostility, and depression, which indicate that the use of different priors affected the classifications of respondents on hostility and alcohol-related problems the most and the least, respectively.

**Fig. 3:**
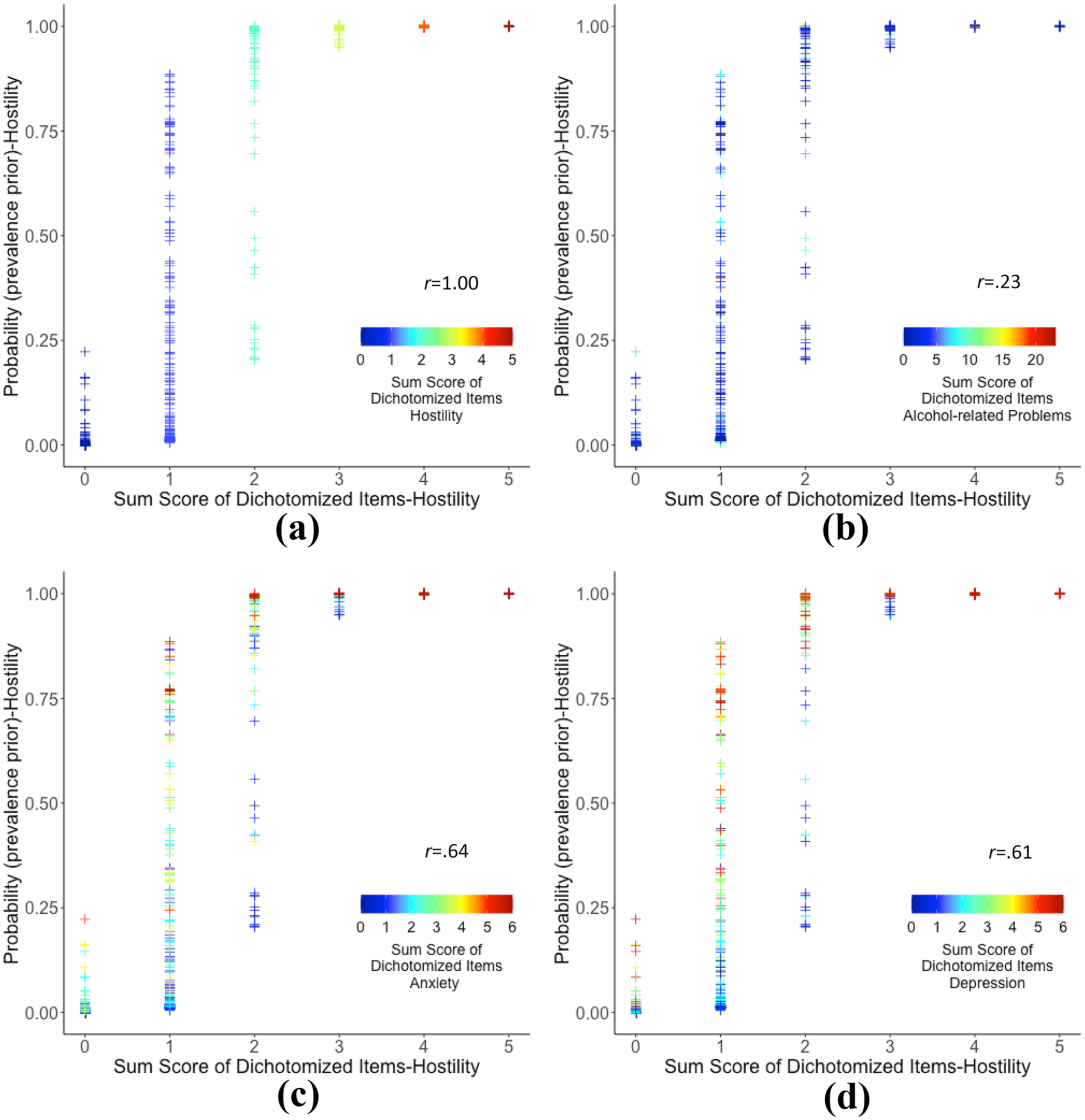
Scatter plots of sum scores of dichotomized hostility items (horizontal axes) against estimated hostility probabilities based on the prevalence prior (vertical axes). From the top left, the color legend indicates the number of items endorsed for hostility (**a**), for alcohol-related problems (**b**), for anxiety (**c**), and for depression (**d**). The correlation coefficient, *r*, between two sum scores of dichotomized items is shown in each plot. Small random noise was added to help distinguish overlapping data points

Figure 3 is provided to better understand how the estimated probability of a mental health problem is related to a respondent’s sum score on the same scale, as well as other scales (i.e., cross-attribute impact). The figure focuses on hostility and represents four of the 16 possible scatter plots that can be generated by examining the four attributes pairwise (see Supplemental Fig. 1 for all the plots). By plotting the estimated probability against the sum score of the same attribute, Fig. 3 demonstrates that respondents who have an identical sum score on a scale can have widely different estimated probabilities. For example, Fig. 3a shows that respondents who have a score of two on the hostility scale can have estimated probabilities that range from .20 to 1.00. It stands to reason that these disparities must have been due to their responses to the other three scales. Upon closer inspection, it can be observed that for a fixed sum score on the hostility scale, higher estimated probabilities were associated with higher sum scores (i.e., warmer colors) on the anxiety and depression scales (Fig. 3c, d), but not necessarily on the alcohol-related problem scale (Fig. 3b). Incidentally, the hostility scale score correlated .64 and .61 with the anxiety and depression scale scores, respectively, and only .23 with the alcohol-related problems scale score. These results underscore the influence of attribute correlations on the estimation of posterior probabilities and how their exclusion from the analysis (i.e., using the independence prior) can lead to potentially disparate respondent classifications.

## Discussion

In research applications, mental health problems and symptoms have commonly been assessed and evaluated using scale scores (e.g., means or totals across items) or latent trait scores derived from factor analysis or IRT models. These severity scores may, in turn, be categorized based on pre-defined clinical cutoffs for diagnostic or screening purposes. However, overall severity score–based approaches may obscure clinically meaningful differences in the presentations of related attributes among individuals with similar scores. The current paper detailed CDMs as an alternative to traditional score-based approaches. CDMs can organize symptom clusters into sets of attributes and attribute profiles, using item-level response data. The CDM approach may be particularly useful when complex heterogeneity in symptoms and attributes exists, which can potentially elucidate different etiological pathways and suggest different prevention and intervention approaches to mental health problems. CDMs may facilitate a richer assessment of attribute presentation that may be obscured when relying exclusively on scale-level severity scores or clinical classifications derived from them.

The shortcomings of categorization and classification have been noted in clinical science as the current nosology may not accurately reflect the dimensional and overlapping nature of many mental health symptoms and disorders, which may be etiologically linked (Haslam et al., 2012); thus, it may be beneficial to simultaneously examine attribute (i.e., symptom cluster or problem) patterns. Similarly, in terms of attribute assessment and classification, additional information from other related symptoms may be particularly helpful. The attribute profiles produced by CDMs accommodate these complex but common scenarios in psychological assessment, including situations consisting of multiple co-occurring symptoms. Thus, the CDM’s granular approach may be particularly well suited as a measurement tool for the transdiagnostic Research Domain Criteria (RDoC) initiative of the National Institutes of Health (Insel et al., 2010).

Lastly, it should be noted that CDMs are functionally similar to multidimensional IRT (MIRT) models that allow for within-item multidimensionalities (Adams et al., 1997), setting aside some technical differences in the latent variables (i.e., continuous vs. discrete). However, the CDM approach offers greater flexibility than its MIRT counterpart. In particular, general CDMs, such as the G-DINA model, subsume a wider range of specific CDMs, including compensatory, noncompensatory, and additive models. As illustrated in this tutorial, the data can inform the specification of the CDM; thus, the specific CDM to be used with an item need not be determined a priori; moreover, an array of CDMs can be leveraged within a single test or survey. In contrast, the specific form of the MIRT model, such as compensatory (Reckase, 1985) or noncompensatory (Sympson, 1978), needs to be determined a priori, with the same model typically adopted for all the items within a test.

### Limitations and Future Directions

Despite the utility of CDMs, several potential limitations need to be considered. First, CDMs require strong domain knowledge and should be theory-based; otherwise, the findings they produce may not be meaningful. In particular, substantive inference from CDMs hinges on a carefully specified definition of the attributes under investigation and their associations with particular items. Thus, items in the assessment inventory should be constructed based on the most current understanding of the symptoms in the specified target population. If any items are extraneous or irrelevant with respect to the attributes of interest, CDMs may not yield meaningful findings.

Second, although the items used in the data example were developed based on specific mental health symptoms, the inventory was not originally constructed following the CDM framework. In principle, the optimal implementation of CDMs would involve formulating items based on the most up-to-date domain knowledge. Developing assessments following the principles of CDMs would allow researchers to include items specifically designed to measure multiple attributes at the same time. However, a ground-up approach is still possible in secondary data analysis applications while avoiding issues of model identifiability (e.g., Gu & Xu, 2021), as demonstrated in the current paper.

Third, the data analyzed in this tutorial were dichotomized from polytomous form for model simplicity and demonstration. Although such dichotomization may involve potential information loss and affect attribute identification, sensitivity analyses using a sequential G-DINA model yielded a similar pattern of results in this specific data application. When larger samples are available and each response category has a sufficiently large number of observations, fitting the data with the sequential G-DINA model may be more appropriate. However, one should note that most of the methodological developments in CDMs are based on dichotomous response models. As such, a number of procedures for polytomous response CDMs, such as item fit evaluation and assessing identifiability, have yet to be developed. It is recommended that researchers carefully consider the theoretical and empirical implications of dichotomization.

Fourth, the data example focused on a specific population and a narrow range of related symptoms; thus, it is possible that the findings may not generalize to other populations. Fifth, the attributes studied in the data example were not validated against other clinical diagnostic criteria, tools or standards, such as a diagnosis made by a trained clinician based on the Structured Clinical Interview for DSM-5 Disorders (SCID-5-CV; First et al., 2016). Thus, validation studies of the application of CDMs in prevention research may be warranted in the future. Finally, the use of CDMs in the assessment of mental health symptoms is not limited to alcohol-related problems, anxiety, hostility, and depression, which we focused on in the current paper. By fully utilizing item-level information, findings from CDMs may provide evidence with a finer resolution to better understand co-occurring latent symptoms that are difficult to assess and quantify. A better assessment is a critical precursor for developing effective prevention and intervention strategies. Therefore, we see CDM as a potentially powerful assessment approach in prevention research.

## Supplementary Information

Below is the link to the electronic supplementary material.

Supplementary file1 (DOCX 728 KB)
