# Supplementary material for: A Tutorial on Cognitive Diagnosis Modeling for Characterizing Mental Health Symptom Profiles Using Existing Item Responses
Source: Prev Sci. 2022 Feb 3;24(3):480–92. doi: 10.1007/s11121-022-01346-8 (PMC10115700; doi:10.1007/s11121-022-01346-8)
Supplement: Supplementary file 1 — Supplementary file1 (DOCX 728 KB) [file 11121_2022_1346_MOESM1_ESM.docx]

**Supplemental Material**

A Tutorial on Cognitive Diagnosis Modeling for Characterizing Mental Health Symptom

 Profiles Using Existing Item Responses

Zhengqi Tan, Jimmy de la Torre, Wenchao Ma, David Huh, Mary E. Larimer, Eun-Young Mun

**Technical Supplement**

**Model Formulation of CDMs**

The binary attribute profile can be denoted by $\boldsymbol{\alpha}_{l}=$ ${\{\alpha}_{lk}\}$, where *k* = 1, …, *K* indicates the specific attribute and *l* = 1, …, $2^{K}$ the specific attribute profile.

Mathematically, the Q-matrix is a *J* × *K* design matrix of ones and zeros, where each row corresponds to one of the *J* items, and each column corresponds to one of the *K* attributes. The *jk*^th^ element of the matrix, *q_jk_*, indicates whether the *k*^th^ attribute is measured by the *j*^th^ item (= 1) or not (= 0). Consequently, each item has its own q-vector ***q****_j_*, which indicates the relevant attributes for the item.

For notational convenience, we can let the first $K_{j}^{*}$ attributes be the required attributes for item *j*, and $\boldsymbol{\alpha}_{lj}^{*}$, *l* = 1, …, $2^{K_{j}^{*}}$, the reduced attribute profile pattern consisting of the columns of $\boldsymbol{\alpha}_{l}$ required by item *j.* In general, $K_{j}^{*}=\sum_{k=1}^{K} q_{jk}$. The item response function of the G-DINA model describing the probability that a respondent with a reduced attribute pattern $\boldsymbol{\alpha}_{lj}^{*}$will endorse item *j*, using the identity link, can be written as,

$P\left( \boldsymbol{\alpha}_{lj}^{*} \right)=\delta_{j0}+\sum_{k=1}^{K_{j}^{*}} \delta_{jk}\alpha_{lk}+\sum_{k^{'}=k+1}^{K_{j}^{*}} \sum_{k=1}^{K_{j}^{*}-1} \delta_{jkk^{'}}\alpha_{lk}\alpha_{lk^{'}}+\ldots+\delta_{j12\ldots K_{j}^{*}}\prod_{k=1}^{K_{j}^{*}} \alpha_{lk}$,

where $P\left( \boldsymbol{\alpha}_{lj}^{*} \right)$ denotes the probability of item endorsement;$\delta_{j0}$ is the intercept term; $\delta_{jk}$ is the main effect of $\alpha_{lk}$; $\delta_{jkk^{'}}$ is the interaction effect between $\alpha_{lk}$ and $\alpha_{lk^{'}}$; and $\delta_{j12\ldots K_{j}^{*}}$ is the highest-order interaction effect due to all attributes.

Supplemental Table 1. *Mapping of Item Numbers in the Current Paper to RAPI and BSI Item Numbers*

| Item Number |  |  | Item Number |  |  |
| --- | --- | --- | --- | --- | --- |
| Current | RAPI | Attribute | Current | RAPI/BSI | Attribute |
| 1 | RAPI 01 | AP | 21 | RAPI 21 | AP |
| 2 | RAPI 02 | AP | 22 | RAPI 22 | AP |
| 3 | RAPI 03 | AP | 23 | RAPI 23 | AP |
| 4 | RAPI 04 | AP | 24 | BSI 01 | AN |
| 5 | RAPI 05 | AP | 25 | BSI 12 | AN |
| 6 | RAPI 06 | AP | 26 | BSI 19 | AN |
| 7 | RAPI 07 | AP | 27 | BSI 38 | AN |
| 8 | RAPI 08 | AP | 28 | BSI 45 | AN |
| 9 | RAPI 09 | AP | 29 | BSI 49 | AN |
| 10 | RAPI 10 | AP | 30 | BSI 06 | HO |
| 11 | RAPI 11 | AP | 31 | BSI 13 | HO |
| 12 | RAPI 12 | AP | 32 | BSI 40 | HO |
| 13 | RAPI 13 | AP | 33 | BSI 41 | HO |
| 14 | RAPI 14 | AP | 34 | BSI 46 | HO |
| 15 | RAPI 15 | AP | 35 | BSI 09 | DE |
| 16 | RAPI 16 | AP | 36 | BSI 16 | DE |
| 17 | RAPI 17 | AP | 37 | BSI 17 | DE |
| 18 | RAPI 18 | AP | 38 | BSI 18 | DE |
| 19 | RAPI 19 | AP | 39 | BSI 35 | DE |
| 20 | RAPI 20 | AP | 40 | BSI 50 | DE |

*Note.* RAPI = The Rutgers Alcohol Problems Index, BSI = The Brief Symptom Inventory, AP = Alcohol-related problems, AN = Anxiety, HO = Hostility, DE = Depression.

Supplemental Table 2. *The Concordance of the Attribute Profiles Estimated from the Sequential G-DINA Model with Polytomous Responses and the G-DINA Model with Dichotomized Responses*

|  | At least one attribute is identical | At least two attributes are identical | At least three attributes are identical | | All four attributes are identical |
| --- | --- | --- | --- | --- | --- |
| At least 25 per category | 1.00 | .99 | .95 | .80 | |
| At least 50 per category | 1.00 | .99 | .97 | .84 | |

*Note*. In the sequential G-DINA model, response categories were combined to ensure each category had *n* responses, where *n* had two levels: 25 and 50.


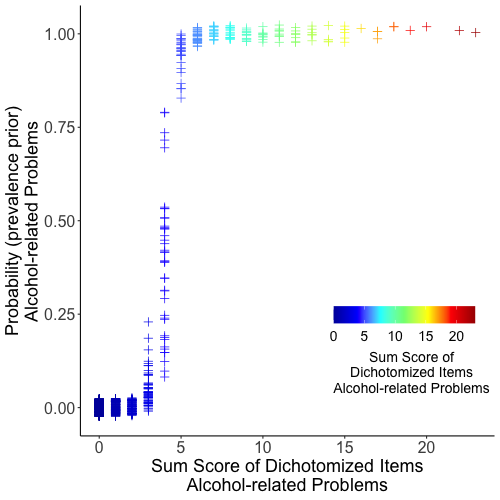

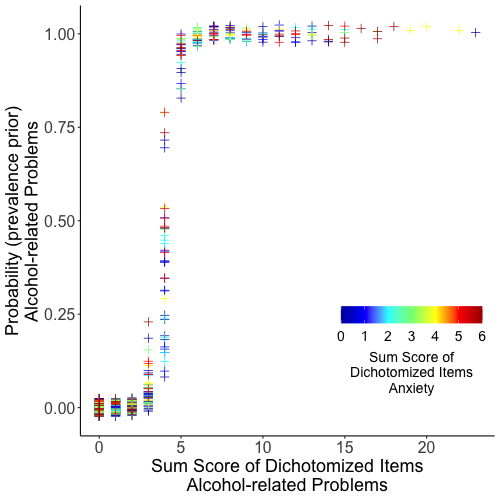

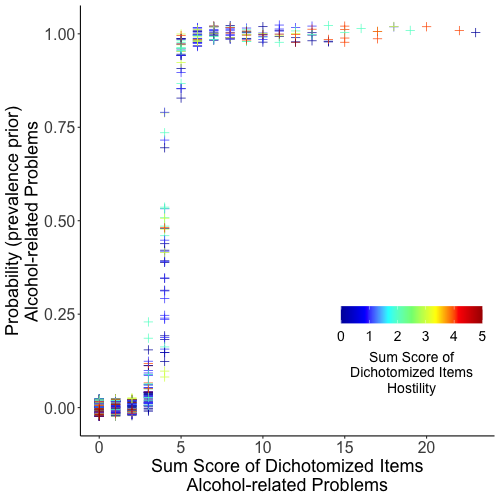

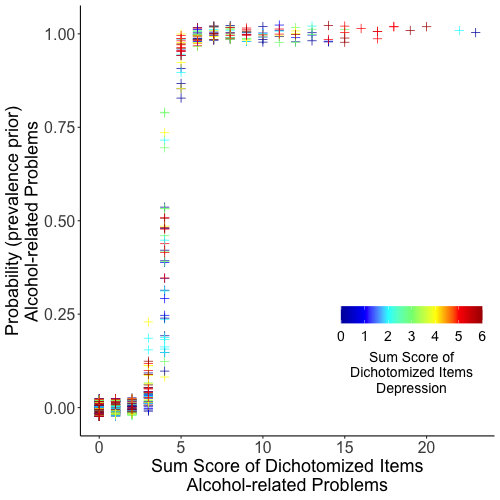

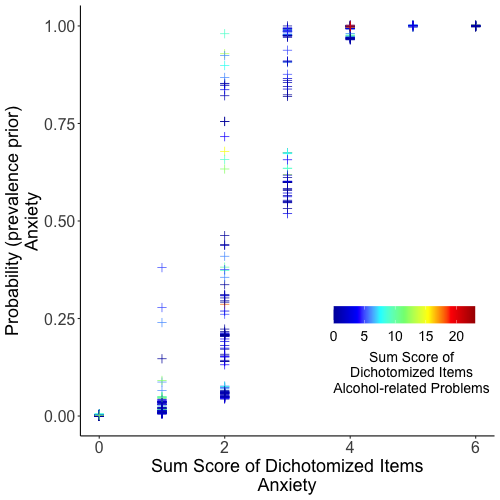

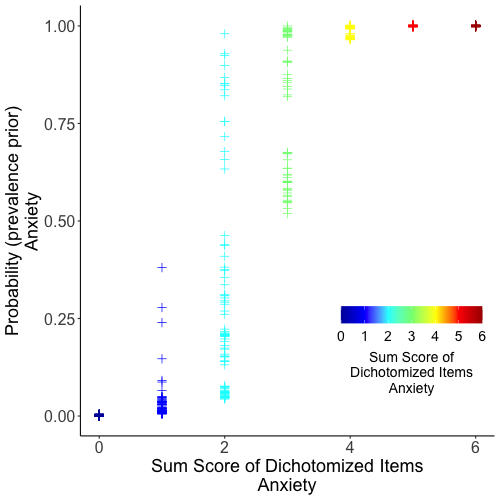

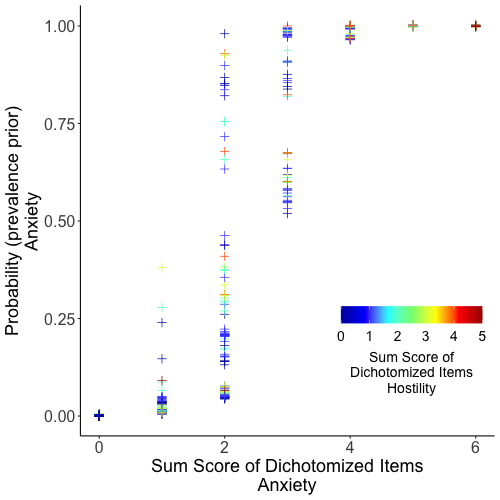

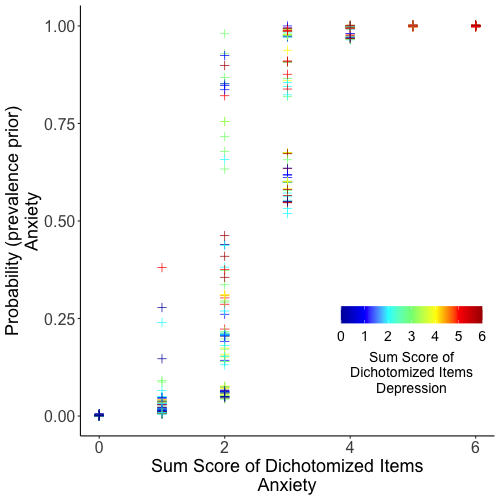

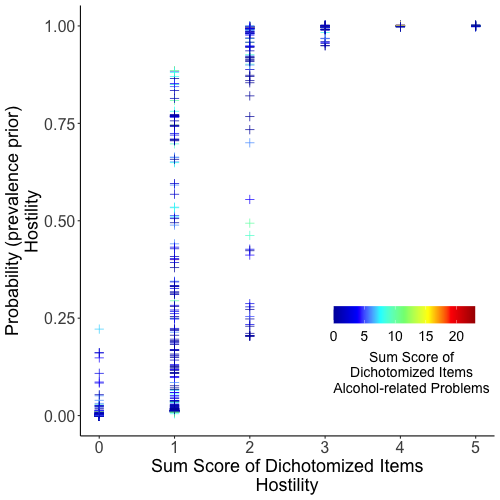

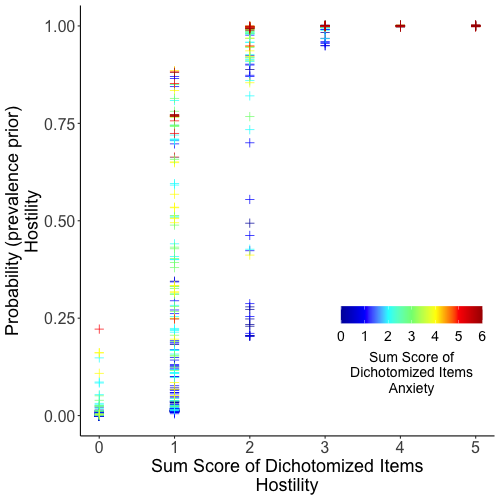

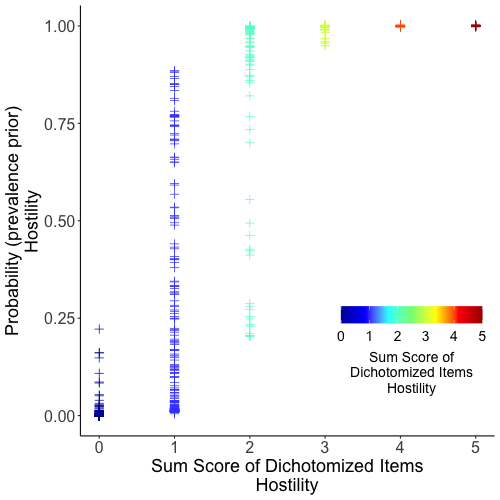

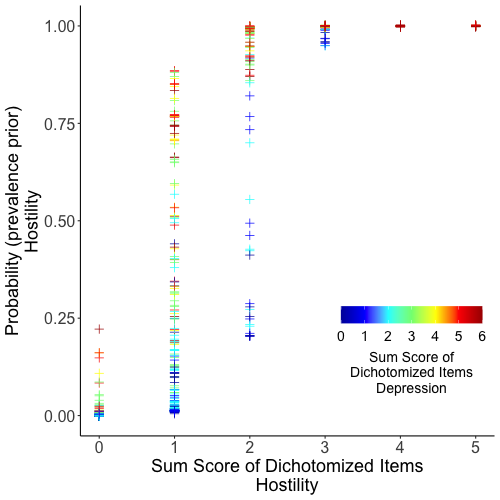

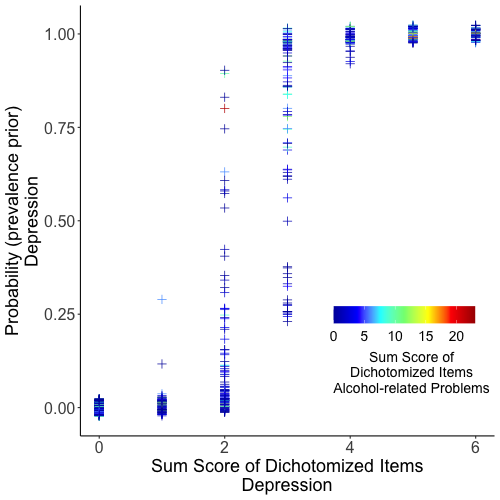

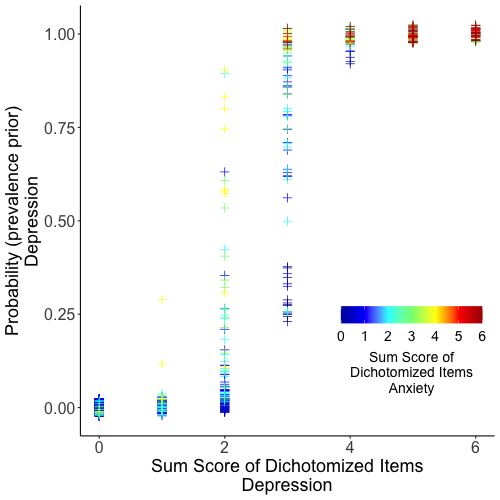

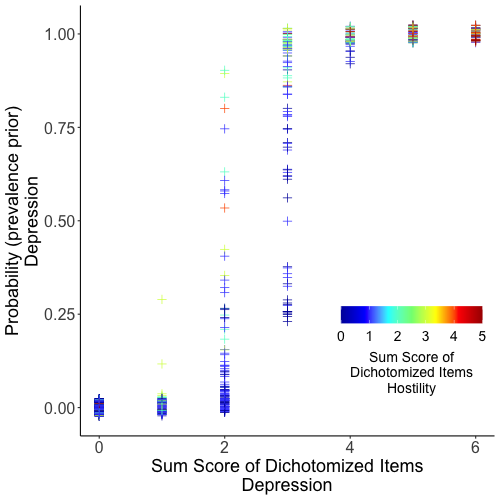

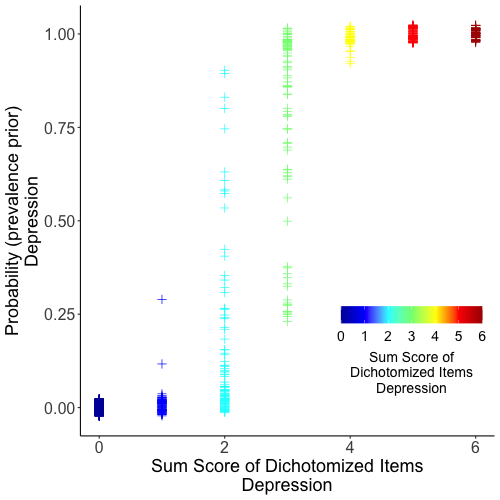


*r*=.24

*r*=.23

*r*=.64

*r*=.24

*r*=.71

*r*=.61

*r*=1.00

*r*=1.00

*r*=1.00

*r*=1.00

*r*=.61

*r*=.71

*r*=.24

*r*=.23

*r*=.64

*r*=.24

Supplemental Figure 1. Sum scores of the dichotomized items (horizontal axes) by the estimated attribute probabilities based on the prevalence prior (vertical axes) for Alcohol-related problems (top row), Anxiety (second row), Hostility (third row), and Depression (fourth row). The color legend indicates the number of items endorsed within each attribute by column: Alcohol-related problems (first column), Anxiety (second column), Hostility (third column), and Depression (fourth column). The correlation coefficient *r* between two sum scores of dichotomized items is shown in each plot. Small random noise was added to distinguish overlapping data points.
